# 29^th^ Annual meeting of the Society for Immunotherapy of Cancer (SITC)

**DOI:** 10.1186/s40425-015-0062-4

**Published:** 2015-05-19

**Authors:** Arthur A Hurwitz, Sylvia Lee, Susan Knox, Holbrook Kohrt, Gregory Verdeil, Emanuela Romano, Kim Margolin, Walter J Urba, Daniel E Speiser

**Affiliations:** National Cancer Institute, Frederick, Maryland, USA; University of Washington, Seattle, Washington, USA; Fred Hutchinson Cancer Research Center, Seattle, Washington, USA; Stanford University, Stanford, California, USA; Stanford Cancer Institute, Stanford, California, USA; Ludwig Center for Cancer Research, Lausanne, Switzerland; Service of Medical Oncology, University of Lausanne, Lausanne, Switzerland; Earle A. Chiles Research Institute, Providence Cancer Center, Portland, Oregon, USA

**Keywords:** Immunotherapy, Cancer, Adaptive immunity, Innate immunity, Adoptive immunotherapy, Immune escape, Checkpoint inhibitors, Tumor immunity, Immune suppression

## Abstract

The 29^th^ annual meeting of the Society for Immunotherapy of Cancer (SITC) was held November 7-9, 2014 in National Harbor, MD and was organized by Dr. Arthur A. Hurwitz (National Cancer Institute), Dr. Kim A. Margolin (Stanford University), Dr. Daniel E. Speiser (Ludwig Center for Cancer Research, University of Lausanne) and Dr. Walter J. Urba (Earle A. Chiles Research Institute, Providence Cancer Center). This meeting included over 1,600 registered participants from 28 separate countries, making it the largest SITC meeting held to date. It highlighted significant worldwide progress in the development and application of cancer immunology to the practice of clinical oncology, including advances in diagnosis, prognosis and therapy, utilizing several immunological pathways and mechanisms for a variety of oncologic conditions. Presentations and posters demonstrated that many concepts that had been pursued preclinically in the past are now being translated into clinical practice, with clear benefits for patients.

## Meeting summary

The 29^th^ annual meeting of SITC took place at the Gaylord National Hotel and Convention Center. It was an exciting meeting marked by a number of extraordinary achievements in the field of tumor immunology and immunotherapy. The gathering came in the wake of great news about major progress in clinical cancer immunotherapy, with promising results of anti-PD1 and anti-PD-L1 therapy in advanced melanoma and further solid tumors. Dr. Finn gave a keynote lecture entitled: “Prophylactic Cancer Vaccines - Feasibility and Progress”. Dr. Trinchieri of the National Cancer Institute received the 2014 Richard V. Smalley, MD Memorial Award for his outstanding research and achievements in cancer therapy. As part of the award, he gave a keynote address entitled, “Cancer as a Disease of the Metaorganism.” In this report, we summarize the sessions of the meeting. Slides from many presentations are available on the SITC website for meeting attendees and SITC members.

### NCI update

William Merritt (National Cancer Institute) gave a grant funding update and discussed current and pending Program Announcements as well as updating NCI-held immune modifiers and the NCI Experimental Therapeutics Clinical Trials Network (ETCTN). The total appropriation to NCI has remained fairly constant since 2009 while the number of research project grants (RPGs) declined following a peak in 2008 (5,472) to 4,816 in 2013. The success rate for RPGs has declined from 23.6% in 2004 to 13.6% in 2013. Just about all grants in the top 9% were funded, some between the 9^th^ and 15^th^ percentiles, and a few grants between the 15^th^ and 25^th^ percentiles were funded by “Exception”. Dr. Merritt indicated that no significant changes are expected in the current fiscal year (FY15).

A new opportunity to collaborate with NIH scientists and the clinical center was presented. Opportunities for research on “Metabolic Reprogramming to Improve Immunotherapy”, “Pathway to Independence Awards in Cancer Research”, and the “Transition Career Development Award” were described. Dr. Merritt also announced the upcoming PAs/RFAs for “Assay Validation for High Quality Markers for NCI-supported Clinical Trials” and he indicated that another release of a Provocative Questions RFA is planned for Spring 2015. The new NIH Genomic Data Sharing policy was also described.

The inventory of NIH-held immune modifiers was described with an emphasis on IL-15, IL-7, IL-12, and anti-PD-1 (both nivolumab and pembrolizumab). The contacts for investigators interested in obtaining these agents for clinical trial can be found in the slide deck on the SITC website. These and other checkpoint blockade inhibitors may also be available for protocol development in the ETCTN, whose objectives were discussed by Dr. Merritt. The major theme is to encourage Team Science and collaborations between the extramural and intramural communities. The major goals are as follows: 1) Research and development for new treatments; 2) Tumor characterization in biomarker-driven studies; 3) Enhanced understanding of cancer biology; and 4) Education and training for young investigators.

### FDA update

Raj Puri (U.S. Food and Drug Administration) gave an update on FDA regulatory policy related to cancer immunotherapy. The respective offices within FDA responsible for different oncology products (small-molecules, biologics, cellular and gene therapies, vaccines, etc) were reviewed. The oncology product approvals by the CBER Office of Cellular, Tissue and Gene Therapies (OCTGT) were listed as follows; 1) antigen-presenting cells pulsed with GM-CSF-PAP in androgen-resistant prostate cancer (sipuleucel-T); 2) intravesical therapy for bladder cancer; and 3) hematopoietic progenitor cell therapy using cord blood. Citations and links were given for several recent “Guidance for Industry” documents relevant to immunotherapy including preclinical assessment of investigational cellular and gene therapy products and trial design for studies of virus or bacteria-based gene therapy and oncolytic products. Also discussed were workshops on standards for cellular therapy and regenerative medicine, innovations in breast cancer drug development and courses to train clinical investigators. Breakthrough therapy designation was also discussed. Dr. Puri reported that at the time of the meeting, approximately 30 such requests had been granted. Links to FDA websites, documents and contact information can be obtained from the slides, which are archived on the SITC website.

### Richard V. Smalley, MD memorial award

In 2014, Giorgio Trinchieri was the recipient of the Richard V. Smalley, MD Memorial Award. The Award is given in memory of Dr. Smalley, who was a past President and Charter member of SITC. Dr. Trinchieri (National Cancer Institute, National Institutes of Health) has made seminal contributions to our understanding of innate resistance to cancer and molecular mechanisms of immune cell activation. Dr. Trinchieri delivered a seminar on his recent, ground-breaking work identifying a role for the regulation of tumor immunity by the commensal microbiota. The talk was entitled, “Cancer as a Disease of the Metaorganism”.

Among their initial findings was that depletion of the intestinal microbiota by treatment of tumor-bearing mice with a cocktail of antibiotics reduced the efficacy of an immune-based therapy, which delivered intra-tumoral blockade of IL-10 in combination with the TLR9 agonist, CpG oligodeoxynucleotides. Similar results were also noted using classical platinum-based chemotherapeutics. A role for pro-inflammatory cytokines like TNF and IL-12 were demonstrated for the immune-based therapies, whereas in the chemotherapy model, a strong role for reactive oxygen species was reported, which was associated with NOX2-expressing myeloid cells. These reactive oxygen species mediated DNA adduct formation induced by the platinum-based compounds. While the precise mechanism by which the biome primes myeloid cells is unclear, a role for biome diversity in generating “systemic inflammatory tone” was suggested. The composition of the biome was related to the overall production of TNF and particular species can have positive or negative influences. Oral administration of LPS restored the anti-tumor response in antibiotic-treated animals. While platinum-based compounds required MyD88, the precise receptor-ligand pair remains unknown. Not surprisingly, IFN-based signature pathways also played a critical role in the biome-driven anti-tumor responses. In closing, Dr. Trinchieri shared two images: a nicely maintained park and a forest fire. He used them to suggest that the on-going war against cancer be thought of as a careful system of “park management”, rather than the historical “slash-and-burn approaches”. Clearly, by understanding the complexity of the interaction between the microbiota and immune system, the human metaorganism, as Dr. Trinchieri labeled it, can be manipulated to generate more potent anti-tumor immunity.

### Inflammation, innate immunity, and microbiome

Following the Smalley Award lecture, a symposium entitled “Inflammation, Innate Immunity, and Microbiome” was held. The first speaker was session Co-Chair William J. Murphy from the University of California. Dr. Murphy spoke on the vast and complex biology of NK cells. Dr. Murphy addressed the role of NK cell regulation by cytokines like TGF-β and regulatory T cells. Understanding this process is critical as NK cells contribute to tumorigenesis at multiple stages. Similar to tumor immunity, the anti-viral response is controlled by NK cells. In turn, NK cells are regulated, or “licensed”, by inhibitory receptors that bind to MHC on target cells. This is critical for tumor immunity, as many cancer types down-regulate MHC as an immune evasion strategy, which might make them more susceptible to NK cells. In the mouse, these receptors are marked by Ly49 allotypes. Dr. Murphy provided data demonstrating that in a murine CMV model, particular subsets of Ly49-licensed NK cells were critical for maintaining anti-viral immunity. In the non-transplant setting of leukemia, licensed NK cells did not play a critical role in survival of the host. In contrast, with hematopoietic stem cell transfer, unlicensed NK cells were important for improved survival, with a response comparable to Treg cell depletion. Given the predominance of early NK cell repopulation after stem cell transfer, understanding the mechanisms by which NK cells contribute to engraftment and tumor control will undoubtedly improve cancer therapies and transplant survival.

Session Co-Chair Marco Colonna (Washington University School of Medicine) presented his laboratory’s work on innate lymphoid cells (ILCs). This compartment is comprised of at least 3 known subsets (ILC1, 2, and 3) that have unique functional and phenotypic characteristics, which he likened to helper T cell subsets. ILC3 are NKp44 + CD56 + cells, which are found in multiple tissues. Like Th17 cells, they make IL-22, which triggers antimicrobial peptide synthesis and chemokine expression. These cells were shown to be dependent on the expression of the aryl hydrocarbon receptor for development, and in their absence, mice have altered development of intestinal lymphoid tissues that made them more susceptible to bacterial infections (for example, *C. rodentium*) and resistant to inflammatory bowel disease. ILC1 are NKp44 + CD103+ cells which are elevated in Crohn’s disease and display a TGF-β expression signature. While they share some similarities with NK cells, ILC1 seem to be a divergent cell population with unique function and expression pattern. Dr. Colonna finished by sharing some comparative genetic profiling studies using the ImmGen database. Varied expression profiles enabled comparison of ILC to NK cells in the intestinal tissues and ILC2 to alternatively-activated macrophages. Overall, these studies provided a novel perspective on a unique class of cells that provide critical support to innate immune responses.

Two short talks were selected for presentation from the submitted abstracts. In the first, Leticia Corrales (University of Chicago) described a role for the DNA sensor STING in eliciting anti-tumor T cell responses. Using the B16 tumor model, Dr. Corrales reported that synthetic STING ligands could promote regression of these established transplantable melanomas, with similar results in other tumor models. In the second talk, Ayelet Sivan (University of Chicago) described a role for the microbiome in tumor growth. Different colonies of mice were shown to have different tumor growth kinetics, associated with different T cell activation profiles. Interestingly, fecal transfer from the mice, which support tumor immunity to the mice with faster tumor growth resulted in increased T cell frequencies and slower tumor growth. Ms. Sivan postulated that this may be due to effects on the antigen presenting cell populations. These findings are consistent with those presented by Dr. Trinchieri and should lend themselves to important advances in eliciting tumor immunity.

The final presentation of the session entitled “The role of the intestinal microbiome in GVHD”, was delivered by Marcel van den Brink (Memorial Sloan Kettering Cancer Center). Studies on the role of the microbiota affecting bone marrow failure date back to the 1970’s. Treatment of patients with antibiotics has been observed to improve bone marrow graft acceptance and reduce GVHD, with a similar effect demonstrated in experimental murine models. Conversely, development of GVHD was shown to be associated with a focused intestinal microbiome; poor outcome in bone marrow transplantation was associated with reduced diversity of intestinal flora. Protection from GVHD was associated with the presence of high levels of the genus Blautia, a Gram-positive anaerobic bacterium, with a strong predictive power. Antibiotics reduced the presence of Blautia in patients, as did reduced caloric intake. Further studies demonstrated that in a murine model of GVHD, introduction of Blautia improved survival. From a mechanistic standpoint, bacterial production of short chain fatty acids such as butyrate and propionate improved GVHD outcome. Options for dietary manipulation to increase fatty acid levels were proposed to improve bone marrow transplantation survival. Similarly, antibiotics that spare Blautia were demonstrated to have similar effects. The findings reported by Dr. van den Brink supported the concept that complex interactions between the microbiome and the immune response influence cancer and immune reconstitution. In this case, it appears that the biome components (contributed by Blautia) promote an immune suppressive environment that reduces GVHD and improves survival.

### Lung cancer: immunology and immunotherapy

In the past few years, advances in immunotherapy have transformed our approach to lung cancer treatment. It is now clear that PD-1 immune checkpoint blockade may substantially help lung cancer patients, and there is a new sense of hope in the field. Because lung cancer is the leading cause of cancer deaths worldwide, the sheer numbers of patients who may benefit from these agents has, in turn, transformed the field of immunotherapy itself into a mainstream cancer treatment, rather than a boutique therapy for a few selected patient groups. The session on lung cancer included reviews of the recent advances in clinical development of PD-1/PD-L1 targeted therapies, as well as ongoing work in vaccine therapy and manipulating the tumor microenvironment.

Laurence Zitvogel (Institute Gustave Roussy) described the use of autologous dendritic cell-derived exosomes (Dex), loaded with desired peptides, as a cell-free vaccine strategy to enhance NK cell and T cell anti-tumor immune responses. She presented data from a phase II trial testing metronomic cyclophosphamide followed by Dex, pulsed with MAGE-1, MAGE-3, NY-ESO-1, and MART-1 peptides, as maintenance immunotherapy in HLA-A2 positive patients with advanced NSCLC after disease control with cisplatin-based chemotherapy. In 22 treated patients, the median overall survival was 15 months and time to progression 4 months. While there was minimal induction of antigen-specific T cell or antibody responses, an increase in NKp30 mediated function, measured by TNF-α release, was observed in patients after 4 Dex vaccines and correlated with overall survival. This evidence of immune activity supports further investigation of Dex, perhaps in combination with PD-1 immune checkpoint blockade or other immunomodulatory strategies, ultimately taking into account the molecular characteristics of different subsets of lung cancer and defining biomarkers of immune response and resistance.

Roy Herbst (Yale Cancer Center) reviewed the key findings and challenges to date of PD-1/PD-L1-based immunotherapy in NSCLC. A comparison of the reported data from 5 different anti-PD-1/anti-PD-L1 agents revealed a consistency in response rates at approximately 20% in unselected NSCLC patients. However, this does not capture the true clinical benefit of these agents. The unique patterns of clinical responses to immune checkpoint blockade, including the prolonged durability of many responses, and the pattern of pseudoprogression seen in 10-15% patients, demonstrates a clear need to establish more reliable endpoints for evaluating the benefits of immunotherapy than standard tumor measurements such as the RECIST system. One proposed option is to create a measure of aggregate clinical activity that reflects median duration of response or to use 1-year or 2-year landmarks to capture the long-term benefit of these agents, including prolonged stability or minor regressions. It remains unclear whether any of the structural design differences in the PD-1 and PD-L1 antibodies (with variations in binding affinity, targets, antibody isotype and ADCC potential) will be critical to identify the agent(s) among many current contenders that possesses the optimal therapeutic index, which is likely to be different depending on tumor type and characteristics of the immune tumor microenvironment. Antibodies targeting PD-L1 instead of PD-1 may decrease toxicity because this allows PD-L2 to retain its role in protection against autoimmunity—a concept supported by the observation of fewer events of severe pneumonitis among anti-PD-L1 agents to date; Dr. Herbst cautioned against drawing premature conclusions, since these drugs arrived later, when the clinic was more prepared to handle new immune toxicities, and the outcomes are not fully available yet.

John Nemunaitis (Mary Crowley Medical Research Center) presented phase 2b results from the TIME study of TG4010 immunotherapy combined with first line chemotherapy in patients with advanced NSCLC. TG4010 is a modified vaccinia vector encoding the entire tumor antigen MUC1 and interleukin-2. Retrospective subgroup analysis of earlier phase 1 data suggested a survival advantage from the addition of TG4010 to chemotherapy in patients with a normal level of circulating activated NK cells (CD16+ CD56+ CD69+) but not in patients with a high level. This led to the design of a prospective study, using Bayesian analysis that incorporates the earlier results, to enroll patients with advanced MUC1-expressing NSCLC to receive TG4010 versus placebo with first line platin-based chemotherapy. On subgroup analysis of 221 patients, those with a normal level of pre-treatment activated NK cells (n = 170) and the patients with nonsquamous histology (n = 195) demonstrated a statistically significant improvement in PFS with a trend towards OS benefit. The PFS benefit was greatest in patients designated with both a low level of activated NK cells and nonsquamous histology (n = 152) at 5.9 versus 4.9 months for the TF4010 versus placebo arm, respectively. The vaccine was well tolerated, and a large phase III registrational trial is now underway to confirm and further quantitate the activity of the vaccine in combination with chemotherapy.

Julie Brahmer (Sidney Kimmel Comprehensive Cancer Center) reviewed recent efforts to determine biomarkers and characteristics of response to PD-1 immune checkpoint blockade in NSCLC patients. To date, only two markers have been found to correlate with higher response rates: PD-L1 expression and smoking status. However, neither is reliable in guiding which patients should be treated with these agents as there are many patients whose tumors are PD-L1 negative or are never-smokers who respond to PD-1 blockade as well. The limitations of PD-L1 status include heterogeneity of PD-L1 expression between primary site and metastasis, between metastatic sites of the same tumor, and among cells of the tumor microenvironment. Further, interpretation of these data may be confounded by the induction of PD-L1 expression by the secreted products of local tumor-infiltrating lymphocytes, which is an important determinant of its role as a therapeutic target. In addition, the varying methodologies of PD-L1 testing used by separate academic and industry groups continues to be problematic, because different antibodies and staining conditions are used, with variations in designation of “positive” or “negative” results. For example, the threshold for “PD-L1 positive” is arbitrarily set at 1% by some studies and at 5% by others. Both Dr. Brahmer and Dr. Herbst spoke in favor of standardizing this and further parameters of the assessment of PD-L1 expression. Former and current smokers have higher response rates to PD-1 checkpoint blockade than never-smokers, which is likely due to the increased number of mutations in tumors that result from long-term tobacco exposure. Other clinical characteristics that have been examined but *not* shown to correlate with response include age, sex, performance status, and even histology, despite earlier evidence suggesting the squamous cell histology would have a higher response rate. Biomarkers that failed to show a correlation include tumor-infiltrating lymphocytes, PD-L2 expression, CD4: CD8 ratio, lymphoid aggregates, and necrosis. Dr. Brahmer also commented on future combinatorial strategies to increase the response rates to PD-1 therapy by turning non-inflamed, PD-L1 negative tumors into immune-responsive tumors, through the use of radiation, molecularly targeted therapy, tumor-based vaccines, T cell therapy, and epigenetic priming. It is likely that the optimal strategies for enhancing the outcome of PD-1-directed therapies for lung cancer will differ from those for other tumor histologies.

Mikael Pittet (Harvard University) described work by his group and others examining the role of tumor-associated macrophages (TAMs) that may promote tumor growth. The density of TAM correlates with decreased survival in various cancer types, including NSCLC. Overproduction of angiotensin II (AngII) in a lung cancer mouse model (KP mice) increased hematopoietic stem cells, which increased monocyte precursors to TAMs. Tumors were shown to produce angiotensinogen (Agt), which is a necessary precursor of AngII. Blocking AngII signaling in KP mice with enalapril, an angiotensin-converting-enzyme inhibitor (ACE inhibitor; a drug that is frequently used to treat hypertension), led to a decrease in TAMs, a decrease in tumor growth, and increased survival. These findings are intriguing in light of two retrospective studies that showed improved outcomes in NSCLC patients who were on an ACE inhibitor for treatment of their hypertension [1,2]. These results support a promising new therapeutic direction for targeting tumor-immune cell interactions that may impact on anti-tumor immune responses. A number of other strategies to characterize and manipulate tumor-associated macrophages are ongoing and likely to complement other therapeutic modalities.

### Tumor microenvironment and immunosuppression

A session entitled “Tumor Microenvironment and Immunosuppression” delivered 3 diverse plenary talks and one shorter talk, all describing immune regulatory mechanisms related to the tumor microenvironment. In the first presentation, Suzanne Ostrand-Rosenberg (University of Maryland, Baltimore County) described a novel signaling pathway for the PD-1/PD-L1 axis. In addition to PD-1, CD80 is a second PD-L1 ligand. The binding affinity of CD80:PD-L1 is comparable to CD80:CD28 and PD-1:PD-L1 binding. Tumors that were transduced to express CD80 had reduced PD-1:PD-L1 binding and provided improved T cell priming *in vitro*. A soluble form of CD80 provided similar benefits: increased costimulation of T cells *in vitro* and improved immunity to tumor challenge. Taken together, these findings suggest that providing soluble forms of CD80, either alone or in combination with other immunomodulatory therapies, may be more effective than antibodies directed against PD-1 or PD-L1.

In the second presentation, Gabriel Rabinovich (Instituto de Biologia y Medicina Experimental (IByME)) described the role of Galectins in promoting immune suppression. Galectins are a family of highly conserved secreted lectins, which bind N-acetyl-lactosamine and contribute to protein sorting and turnover. Galectin levels in tumors associate with tumor progression and metastasis, and blocking Galectin-1 promotes T cell-mediated tumor regression. Galectin-1 also promotes immune suppression, in part through regulatory T cells and tolerogenic dendritic cells and their secreted cytokines. Ligands that bind Galectin-1 include glycans and VEGFR2, which are induced by hypoxia on tumor cells and endothelial cells through an NF-κB-dependent process. As such, Galectin-1 binds VEGFR2 and induces angiogenesis; studies reported by Dr. Rabinovich suggest that reducing Galectin-1 levels can improve VEGFR-targeted therapies. Results were presented in multiple primary and transplantable tumor models, which demonstrated the utility of a Galectin-1 antibody at targeting tumor vasculature and enhancing tumor regression through immune-mediated mechanisms. These studies demonstrate that Galectin-1 may serve as a critical regulator of tumor progression. Given the multitude of ligands, directly targeting this unique receptor may provide a powerful way to inhibit tumor progression and improve immunity to tumor antigens, either alone or in combination with other immune-based therapies.

The third plenary lecture of the session was delivered by Weiping Zou (University of Michigan) on the complexity and balance of immunity within the tumor microenvironment. Dr. Zou summarized the role of PD-L1 in inducing suppressive signals in the tumor microenvironment. The impact of this pathway was related to the success of recent clinical trials demonstrating the efficacy of PD-1 blockade. The role of both regulatory T cells and suppressive myeloid cells was also discussed. Specifically, the link between myeloid-derived suppressor cells (MDSCs) and ovarian cancer was presented. Interestingly, MDSCs were also suggested to support (ovarian) cancer cell stemness. This was associated with MDSC-induced expression of micro RNA (miR)101. A more detailed mechanism was presented, which implicated the transcriptional repressor CtBP2 which regulates expression of the stem cell-associated genes *OCT3/4, SOX2*, and Nanog. Additional work was presented on a novel role for IL-22 in promoting stemness of colorectal cancer. Those studies reported that CD4^+^ T cells that secrete IL-22 (Th22 cells) accelerated cancer growth in a human tumor xenograft model as well as a tumor spheroid culture model. IL-22 was associated with increased expression of stem cell-associated genes and Histone H3K70 dimethylation, a marker of gene activation; this was confirmed by association of H3K79 binding to similar stem cell-associated gene promoters, which was STAT3-dependent. A correlation between elevated H3K79 levels and poor outcome in colorectal cancer supported these findings. Dr. Zou presented a novel, “3 signal” model for carcinogenesis which integrates genetic modifications, inflammatory signals that impact stemness, and immune suppressive signals that regulate host defense. These findings underscore the critical relationship between diverse inflammatory responses and carcinogenesis and the need to understand further these complexities.

This session also contained a short talk that was selected from the submitted abstracts. Emanuela Romano (University of Lausanne) described a mechanism by which anti-CTLA-4 (ipilimumab) promotes anti-tumor T cell responses. Interestingly, patients with the highest clinical responses to ipilimumab treatment were shown to have elevated levels of circulating CD11c^+^CD16^bright^ monocytes at baseline. These cells were demonstrated to mediate ADCC of regulatory T cells *in vitro* in presence of ipilimumab. The data presented by Dr. Romano also suggested that ipilimumab responders had increased infiltration of tumors by CD16^+^ macrophages compared to non-responders, correlating with fewer tumor-associated Treg cells. These findings are consistent with previously reported data using experimental murine models and will therefore provide guidance for other checkpoint molecule-targeted therapies.

### Immunopotentiation by radiotherapy: mechanisms and opportunities

Jennifer Jones (NIH/NCI Vaccine Branch) presented data on the identification of optimal immunotherapy targets for use with radiation therapy for metastatic tumors in preclinical models. The data presented demonstrate that TIM-1 costimulates iNKT cells and showed synergy with radiation therapy in controlling growth of the metastatic murine 4T1 breast tumor. A new method, nanoFACS, was described for identification and characterization of extracellular vesicle (EV) subsets including exosomes (30-120 nm) and microparticles (80-800 nm) from tumor and immune cells. This new technique has broad applicability for the study of radiation effects on the tumor microenvironment with single exosome/ microparticle analysis providing a window into the dynamic changes occurring in the tumor immune microenvironment. Proof of concept data were presented validating the fidelity of sorting of EV, with the ability to purify and assess the biological activity of EV from the blood of mice treated with radiation and immunotherapy.

Sandra Demaria (New York University) discussed the role of radiation-induced TGF-β activation as an inhibitor of both dendritic cell activation and T cell effector function. TGF-β blockade using an anti-TGF-β antibody (1D11) in two preclinical breast cancer models resulted in a synergistic benefit when combined with local tumor irradiation (RT), with an associated increase in tumor infiltrating lymphocytes (TILs); and loss of therapeutic benefit was observed following depletion of either CD4 or CD8 T cells. In addition, TGF-β blockade increased RT-induced activation of tumor infiltrating dendritic cells (DC), and allowed priming of tumor specific CD8 T cells against several tumor-derived peptide epitopes following RT. Gene expression studies showed upregulation of immune system activation pathway genes when TGF-β blockade was combined with RT. These results provided a compelling rationale for an ongoing trial of RT with the TGF-β neutralizing antibody fresolimumab used at two different concentrations (1 or 10 mg/kg) in patients with metastatic breast cancer (NCTO1401062, PI S. Formenti). Preliminary analysis demonstrated an increase in tumor specific CD8^+^ T cells in some patients during treatment. An example of an abscopal response seen in a treated patient was shown. Prolonged overall survival and progression-free survival in the arm receiving the higher dose of fresolimumab suggest benefit of blocking TGF-β with radiotherapy. Furthermore, the presence of PD-1 (on TIL), PD-L1 and PD-L2 (on tumor and myeloid cells), suggests that the addition of PD-1 blockade to TGF-β neutralizing antibody and RT will provide further benefit. To test this hypothesis, preclinical experiments were performed in 4-T1-bearing mice to assess the potential benefit of combining inhibition of the PD-1 pathway with RT and TGF-β blockade. Addition of anti-PD-1 to this combination regimen resulted in significantly reduced tumor burden and increased survival in mice as compared to RT and TGF-β blockade alone. Altogether, these data demonstrate the promise of using radiation therapy in combination with multiple immunotherapies as an in situ tumor vaccine strategy.

Simon Dovedi (University of Manchester) reported results from colorectal, breast and melanoma syngeneic tumor models demonstrating that fractionated radiation therapy (fRT) consisting of 5 fractions of 2 Gy resulted in significant upregulation of tumor cell expression of PD-L1 *in vivo*. Cellular depletion studies revealed that RT-mediated increase in tumor cell PD-L1 was dependent on CD8+ T cells. Additional data, showed that adaptive upregulation of tumor PD-L1 following fRT is mediated by tumor infiltrating CD8+ T cells producing IFN-γ, and that this adaptive response is restricted to the irradiated tumor site. These results support the hypothesis that this adaptive resistance mechanism protects tumor cells from immune-mediated killing, and explains, at least in part, why RT alone may not generate systemic tumor antigen-specific responses. Treatment with either anti-PD-1 or anti-PD-L1 antibody in combination with fRT improved mouse survival compared to either treatment alone. Abscopal responses were observed, with up to 60% of mice having a complete response to the combination therapy. These mice were also protected against tumor rechallenge by the generation of long-term immunological memory. Importantly, scheduling of administration of the anti-PD-L1 antibody relative to the fRT was important, with the best results obtained when anti-PD-L1 was started (administered 3 time/wk at 10 mg/kg) on day 1 of the fRT. Efficacy of this combined regimen was CD8+ T cell-dependent. These results demonstrate the potential for enhancing the efficacy of fRT through blockade of the PD-1/PD-L1 axis.

David Roberts (CCR, NIH) discussed the role of CD47 signaling on the relative radiosensitivity of tumors and normal tissues. Binding of CD47 to its ligand, thrombospondin-1, affects a number of pathways including Signal Regulatory Protein α (SIRP α) and immune modulation. Data was presented demonstrating that CD47 blockade using an antisense morpholino protected bone marrow in mice treated with both local and total body irradiation. This radioprotection is mediated by protective autophagy, which is a CD47-mediated stress resistance response. In contrast, CD47 blockade in melanoma and fibrosarcoma syngeneic murine tumor models enhanced RT-induced tumor growth delay (TGD), with this effect being dependent upon CD8+ T cells. There was also synergy between CD47 blockade, adoptive CD8 T cell immunotherapy and RT. While CD47 signaling limits T cell activation, CD47 blockade increases CD8 cytotoxic T lymphocyte (CTL) activity, and in combination with RT enhances granzyme B expression. Furthermore, elimination of CD47 in the tumor microenvironment increased RT-associated TGD. Importantly, in human melanomas, there is an inverse relationship between the level of CD47 expression and the number of CD8+ infiltrating T cells, suggesting that these results may be clinically relevant. The differential effect of CD47 blockade in tumor vs. stromal and normal cells provides an opportunity to increase the therapeutic index of radiation therapy, both alone and in combination with immunotherapy. This is based on data showing that in normal tissues, CD47 blockade increases cell survival via nitric oxide and VEGF signaling, enhanced autophagy, and induction of c-Myc and other stem cell factors. In contrast, in tumor cells, CD47 increases tumor cell death by decreasing protective autophagy, inducing resistance to c-Myc regulation, decreasing resistance to innate immunity and enhancing CTL tumor cell killing. In addition, the results presented demonstrate that CD47 is an immune checkpoint inhibitor for T cells, and that blocking CD47 in combination with RT enhances antitumor immunity by directly stimulating CD8+ cytotoxic T cells, with the potential to increase curative responses.

### Immunotherapy biomarkers task force update

Lisa Butterfield (University of Pittsburgh) spoke on behalf of the Task Force. She described the history and background of the formation of the Task Force and the creation of the following four working groups: 1) Immune monitoring assay standardization and validation-update; 2) New developments in biomarker assays and technologies; 3) Assessing immune regulation and modulation systematically (high throughput approaches); and 4) Baseline immunity, tumor immune environment and outcome prediction. The Task Force met at the SITC 2014 meeting and plans were made for a one-day meeting at NIH in 2015, an Immune biomarkers-based SITC Guest Society Symposium at AAI 2015, and for a manuscript describing new state-of-the-art recommendations for publication in JITC in 2015.

### Update: the CITN

**Figure 1:**
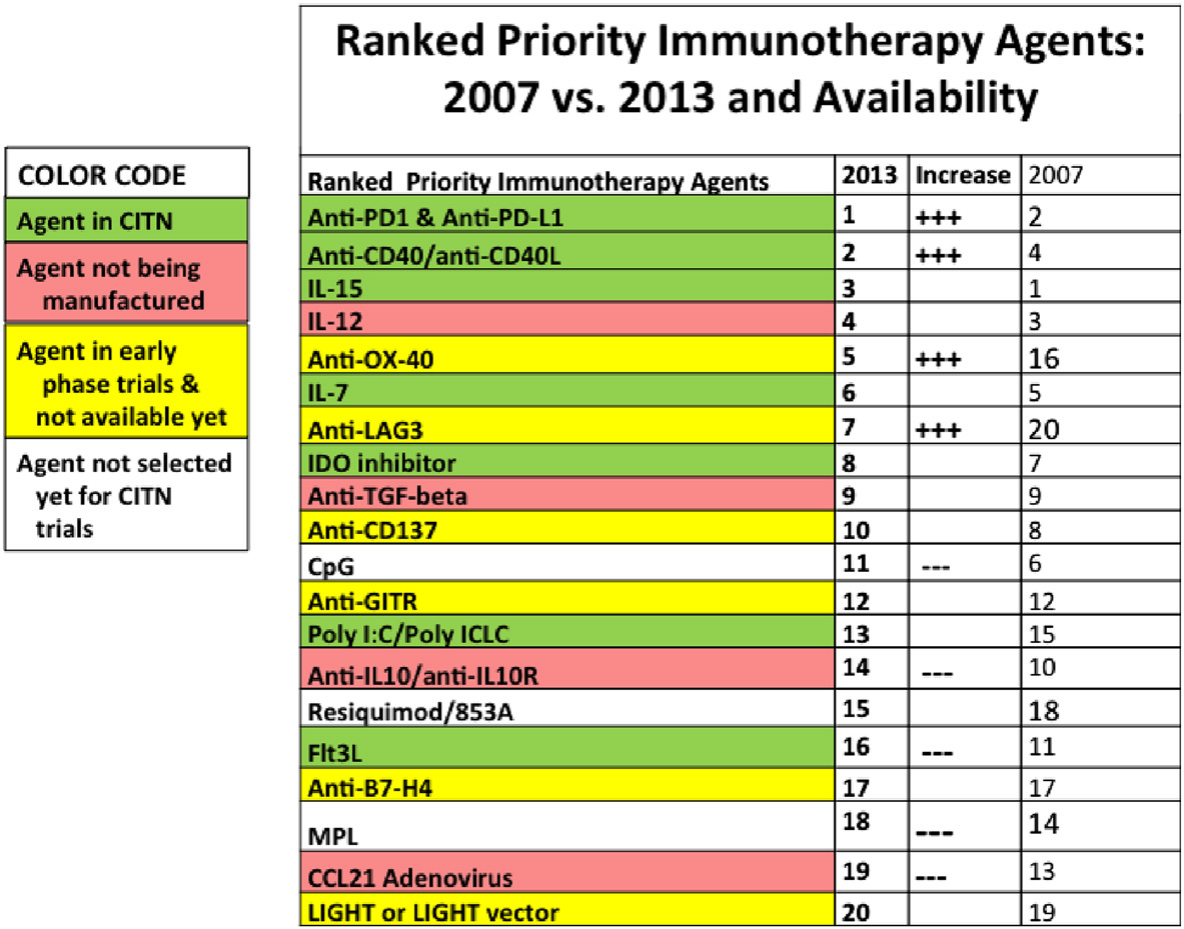
Ranked priority immunotherapy agents: 2007 vs. 2013 and availability.

The Cancer Immunotherapy Trials Network (CITN) continued its success at the mission of making promising experimental agents available to the academic research community, through partnership with the NCI and Industry. Founded in 2011, the CITN experienced growth in 2014, expanding as an NCI-supported consortium from 29 to 32 universities and cancer centers in North America. Bringing together premier cancer immunologists and centers under the direction of PI Martin (Mac) Cheever (Fred Hutchinson Cancer Research Center), the CITN aims to design and conduct early phase trials for patients with cancer, to provide the infrastructure essential for collaboration, and to gain access to top-ranked agents not broadly available for testing. Anti-TIM-3, anti-TNF-alpha, and anti-IL1R were among the recommended additions to the top-ranking agents in 2014. As of the end of 2014, five of the top ten agents were successfully integrated into CITN clinical trials: Pembrolizumab (anti‐PD1) for Merkel cell cancer and Mycosis Fungoides, agonistic anti‐CD40 with neo-adjuvant chemotherapy for pancreatic cancer, IL15 and an immunoconjugate of IL15 with its receptor for NK and CD8 expansion in several solid tumors, IL7 with sipuleucel-T for prostate cancer and as an immunomodulator following adjuvant chemotherapy for older patients with breast or colon cancer, and an inhibitor of indoleamine dioxygenase for ovarian cancer or with a multipeptide vaccine for advanced melanoma (Figure 1). Three priority agents (agonistic anti‐OX‐40, anti‐LAG, and agonistic anti‐CD137) are currently not available but likely to be in 2015, and two agents of interest (IL-12, anti‐TGF-β) are no longer manufactured. Anticipated accrual exceeds 200 patients over ten trials of seven agents, and most of the trials have opened recently, promising a large percentage of accrual over the next year. Each trial is designed to test a critical clinical question and to deliver a large dataset of immunologic correlates that will elucidate mechanisms of action and resistance that will inform future trials, most likely combination strategies. In addition to the above-listed agents in trial, the CITN has recently opened an adjuvant trial for melanoma using the dendritic cell-targeting antibody DEC-205 conjugated to NY-ESO-1 antigen in combination with the Toll receptor 3 agonist polyIC:LC with or without FLT3L for dendritic cell expansion. Preliminary results of the Phase I dose escalation trial of IL-15 were presented at the meeting, establishing safety and immunologic activity and highlighting significant expansion of natural killer cells in circulation [3]. Looking forward, in 2015 the CITN aims to expand to fifteen trials of the priority agents and to focus on investigating the etiology of and potential to reverse resistance pathways to immunotherapy with planned amendments to the ongoing anti-PD1 clinical trials in Mycosis Fungoides and Merkel Cell Carcinoma. By incorporating serial biopsies and “rescue” radiation therapy in patients not optimally responding to PD-1 blockade, the CITN will begin to address the ability of radiotherapy to overcome resistance and induce a response in lesions separate from the radiated site(s) of progression and to better understand the mechanisms and requirements for radiotherapy-induced immune responses (detailed elsewhere in this summary).

### Saturday keynote address

The second Keynote presentation, entitled, “Prophylactic Cancer Vaccines - Feasibility and Progress”, was delivered by Olivera Finn, (University of Pittsburgh). Dr. Finn set the foundation for the talk by describing the public health problems of cancer management, the need for more effective preventive therapies, and the benefits of using prophylactic immunotherapy approaches. Dr. Finn’s success in this arena dates back to 1993, when her first trial of a MUC-1 vaccine was conducted in patients with breast, colon, or pancreatic cancers. In many cancerous tissues, MUC-1 expression loses its polarization and MUC-1 has altered glycosylation. Targeting the hypoglycosylated form was tested using various adjuvants, with only a maximal 20% response rate (as measured by disease stabilization or delayed time to progression). More recently, Dr. Finn’s group has tried to optimize the vaccine and target therapy to “intermediate” stage patients: those with adenoma at the time of detection. An intradermal vaccine showed a 50% response rate as measured by MUC-1-specific IgG, T cell responses, and increased levels of MUC-1-specifc memory T cells. A Phase II trial is currently underway to determine vaccine efficacy. It will enroll ~120 patients at 13 centers and have a follow-up time of up to 5 years. Future studies may enroll cancers of diverse types. An effort is underway to understand the reasons why up to 50% of vaccinated patients do not exhibit a detectable response. One clue may come from the observation that these patients have higher levels of circulating cells that resemble MDSCs. However, no association with age, gender, race, prior viral infections, and HLA haplotype was noted. Moving forward, future studies will also examine the feasibility of MUC-1 serving as an immune target for a variety of diseases and cancer types. By studying the biology and genetics of immune responses to MUC-1, a highly conserved cancer antigen, more effective prophylactic vaccines can be developed.

### Vaccines session

The session on vaccines provided extended insight into the scientific challenges of vaccine development. Hans-Georg Rammensee (University of Tübingen) gave an overview on the “HLA-ligandome”, as it can be identified with mass-spectrometry based approaches to interrogate human tumors. Interestingly, this ligandome is largely different from the transcriptome, with only rare peptides detected in common, probably because gene products have considerably different RNA handling and processing. Not surprisingly, the isolated peptides differ in copy numbers, and this appears to be related to the likelihood of T cell responses. Current and future investigations will clarify how often one can find mutated antigens, relative to the non-mutated antigens that have already been characterized in large numbers.

Craig Slingluff (University of Virginia) and his team are performing multiple clinical vaccination trials in melanoma patients, with the aim to perform side-by-side comparisons of different antigens and adjuvants in a stepwise vaccine development. This group has shown clinical responses in patients who generated strong CD4^+^ T cell responses to vaccination with tumor antigens. In different studies, T cell responses were readily induced despite mixing as many as 12 peptides in the vaccine formulation, showing that potential competition between peptides does not preclude proper T cell triggering. The use of IFA was beneficial for obtaining T cell responses, as opposed to GM-CSF. Careful studies of the vaccine-site microenvironment (VSME) showed induction of chemokines, and T-bet and Gata3 in T cells. Vaccination with the adjuvant AS15, containing CpG oligonucleotides, induced a higher and more favorable Tbet/Gata3 ratio in the VSME.

Jill Slansky (University of Colorado) described the identification of epitopes and related mimotopes in breast cancer. Among tumor-antigen specific TILs, the more frequent TCRs recognized mimotopes that were preferentially associated with anti-tumor immunity, underlining the great interest of molecular characterization of those TCRs. For this, the group used a novel “Emulsion RT-PCR” enabling the combined identification of TCR alpha- and beta-chains. This technique revealed frequent sharing of TCRs between patients, further supporting the concept that naturally occurring TCRs may guide the choice of optimal antigens for immunotherapy.

Gosse Adema (Radboud University Medical Center) presented novel strategies of TME reprogramming, by focusing on gangliosides or sialic acids. The latter dominate the sugars in tumors (in contrast to healthy tissues) and are thus attractive therapy targets. Indeed, inhibitors of sialyl transferases show promising results in preclinical models.

Daniel Speiser (Ludwig Center for Cancer Research of the University of Lausanne) provided an overview on the current progress and challenges in T cell vaccination. Particular emphasis was given to TCR affinity and T cell avidity, which can be favorably promoted by vaccinations with low antigen doses and long intervals (at least 4 weeks) between booster vaccinations. Next generation vaccines (“NGVs”) will likely arise progressively by combining antigens with innate immune stimulators, which need to be tested in small clinical trials for their capabilities to induce T cell responses with high level correlates of protection. These are: 1) high numbers of multifunctional T cells (despite the use of low antigen doses), 2) high avidity T cells, 3) multi-clonality, 4) migration to tumors, 5) broad targeting of multiple tumor antigens, and 6) long-term activity and persistence of responding T cells.

### Late-breaking abstract sessions

Four abstracts were selected for oral presentation in two late-breaking abstract sessions. In the first session, Jennifer Sims (Columbia University) shared her work on TCR repertoires in brain cancer. Using next-generation sequencing of TCR, Dr. Sims reported a unique clonality among tumor-infiltrating T cells in glioblastoma multiforme (GBM) patients as compared to peripheral blood T cells. She also presented data correlating TCR genotypes with gene expression signatures of immune suppression, leukocyte recruitment, and stress responses. In addition, GBM patients treated with anti-CCL2 had reduced TCR diversity. Dr. Sims suggested that TCR diversity can be an indicator of response to therapy and serve as a “metric” for determining individual therapies.

The second talk was delivered by Nina Bhardwaj (Mt. Sinai School of Medicine), who spoke on activation of DCs by matrix metalloproteinase (MMP) 2. Treatment of DCs *ex vivo* resulted in increased expression of OX40L and TNF as well as cleavage of the type I IFN receptor. Activation by MMP2 was TLR2 dependent, and a physical association between MMP2 and TLR2 was demonstrated. MMP2 was also associated with a T_H_2 response following antigen sensitization. In the context of tumors, Dr. Bhardwaj suggested that MMP2 expression may promote immune evasion through supporting type 2 responses.

In the second late-breaking abstract session, Laurent Humeau (Inovio Pharmaceuticals) presented work on a clinical trial employing a DNA-based HPV vaccine for treatment of cervical cancer. Patients (~150) enrolled in the trial had grade 2/3 or 3 cervical intraepithelial neoplasia (CIN), and received 3 doses of an experimental HPV16/18 vaccine delivered by intradermal or intramuscular electroporation. Regression of disease (to CIN1) and HPV clearance was reported to be greater in patients who received the vaccine. Additional analysis of immune parameters was described as underway. The promising results were suggested to be enabling additional vaccine trials.

Finally, Amy Moran (Earle A. Chiles Research Institute) talked about “OX40 agonist immunotherapy expands tumor reactive CD8 T cells and synergizes with PD-L1 blockade to promote tumor regression”. Using Nur77 expression as a surrogate marker for TCR affinity (signal strength), Dr. Moran shared data using an experimental tumor model, which demonstrated that both OX40 agonist and CTLA-4 blockade resulted in Nur77^hi^ T cells infiltrating tumors. OX40 ligation also synergized with blockade of the PD-1 axis, which generated a more robust memory cell population. These findings indicate an effective synergistic therapy, and reveal some important mechanistic insights on how OX40 ligation can promote more durable tumor immunity.

### Oral poster sessions

In the first session, Jieqing Chen (MD Anderson Cancer Center) reported on predictive immune biomarker signatures in the TME of melanoma metastases associated with TIL therapy. With a novel iterative biomarker analysis, a nine-gene gene expression signature was determined by nanostring. Lactoferrin (LTF) was found to be overexpressed in tumors of patients that responded favorably to TIL therapy. In turn, overexpression of IRAK1 (an inflammation driver triggering NF-kB) was found in tumors of non-responders, advocating for using existing and new drugs that block IRAK1. Wenqian Wang (University of Pittsburgh) reported that depletion of HMGB1 in DCs suppressed tumorigenesis and promoted viral clearance. In a mouse model of DC-specific HMGB1 deletion, they showed significantly improved immunity against vaccinia virus and pancreatic cancer using state-of-the-art imaging technologies. Antonia Mueller (University Hospital Zurich) and Holbrook Kohrt (Stanford Cancer Institute) showed synergistic anti-lymphoma activity of rituximab with 4-1BB and PD-1 blockade that can be considered as promising sequential treatments. Similar approaches may also be applied in breast cancer (targeting HER-2) or head and neck cancer (targeting EGFR). Marcin Kowanetz (Genentech) presented data demonstrating circulating and tumor-based biomarkers associated with responses in cancer patients treated with anti-PD-L1 mAb. Besides PD-L1 expression in tumors, responses were associated with high effector T cells and IFN-γ signatures. In blood, IL-18, ITAC, and CD8 + Ki67 + HLADR+ cells were associated with clinical responses. Claire Vanpouille-Box (New York University School of Medicine) presented their work entitled “Fractionated but not single-dose radiation releases key signals of in situ tumor vaccination”. They suggested that fractionated radiation therapy mimics viral infection, with induction of type I IFN, leading to DC mobilization. Such studies are critical for the rational design of clinical trials exploring combination of radiotherapy with immunotherapy.

In the second oral poster session, Rikke Andersen (Herlev Hospital) *et al*. summarized interesting results of treating melanoma patients with expanded autologous TILs. They pursued an approach with intermediate doses of IL-2 allowing most patients to be maintained on the intended IL-2 dose. The clinical results are favorable, confirming that TIL therapy continues to be highly promising, and serves as an excellent basis for future refinement of adoptive cell therapy (ACT). Sonia Guedan (University of Pennsylvania) et al. reported the effects of signaling cassettes introduced into CARs (chimeric antigen receptors). In a carefully designed mouse model, they tested combinations of CAR transduced CD4 and CD8 T cells with regard to their capacities to fight tumors. Their results provide important clues for optimal design of next generation CARs for clinical use. Amanda Harper (The Ohio State University) et al. used a mouse model to demonstrate that MICA expression on monocytes can enhance NK cell responses via NKG2D, leading to enhanced response against antibody-coated tumor targets. Priti Hegde (Genentech) et al. examined responses of bladder cancer patients to anti-PD-L1 treatment and observed that enhanced expression of PD-L1 at baseline was favorable; whereas, abundance of myeloid cells and associated cytokines, particularly Il-1B, was associated with an unfavorable clinical outcome. Lance Miller (Wake Forest School of Medicine) et al. performed extended gene profiling of tumor samples from breast cancer patients, and defined prognostic signatures distinguishing different qualities of tumor microenvironments. This approach allows the screening of dominant molecular pathways, and may be combined with imaging techniques. Finally, Laura Carter (Lycera) et al. introduced novel RORγ agonists and their potency for favorable modification of T cell polarization, promoting Th17 and Tc17 cells, as opposed to Tregs. This approach was suggested to be tested in the clinic soon.

### Presidential session

Numerous factors play a role in the development of an effective antitumor response. This year’s presidential session included presentations that examined important mechanisms by which the production of an effective anti-tumor response can be inhibited. The contributions of myeloid-derived suppressor cells and β-catenin signaling were examined. The role of another coinhibitory receptor TIGIT (T cell immunoglobulin and immunoreceptor tyrosine-based inhibitory motif [ITIM]) was reported. The introduction of CD19-specific modified chimeric antigen receptor (CAR) T cells to treat patients with refractory B-cell malignancies has been a great success. However, translation of this strategy to an effective therapy for solid tumors remains challenging. A study was presented describing the development of a humanized anti-EGFR variant III chimeric antigen receptor, its introduction into T cells, and its preclinical validation, and the initiation of a phase I clinical trial.

A number of attempts have been made to reduce MDSC-induced suppression in tumor-bearing hosts, with only limited impact. Paul Thevenot (Louisiana State University) reasoned that identifying and inhibiting the mediators of MDSC regulatory activity would help overcome T-cell suppression and increase the efficacy of T-cell based immunotherapy of cancer. They attempted to determine whether stress sensor C/EBP-homologous-stress-related protein (CHOP), a downstream product of the integrated stress response, was a master regulator of MDSC-suppressive activity. The ablation of CHOP altered the function of MDSC, decreased their suppressive activity, which allowed the accumulation of CD8 T cells with antitumor activity and impaired C/EBPβ activity and led to a reduced production of the suppressive mediators IL-6 and ARG-1. CHOP was proposed to be a potential target to inhibit MDSC suppressive activity in solid tumors.

Stefanie Spranger (University of Chicago) explored the mechanisms by which tumors are able to prevent infiltration by T cells. Using molecular profiling, they demonstrated that half of tumors that lack a T-cell signature exhibit alterations in the Wnt/β-catenin signaling pathway. Using an autochthonous mouse model of melanoma (inducible BRAF V600^E^ and PTEN deletion) that was also capable of inducible expression of β-catenin, they showed that T-cell infiltrates were universally absent in tumors that expressed β-catenin. Tumors with no β-catenin expression contained infiltrates of T cells, albeit with exhausted phenotypes, that responded to combined therapy with anti-CTLA-4 and anti-PD-1. Tumors that expressed β-catenin did not develop T-cell infiltrates and did not respond to combination immunotherapy, unless a deficiency of tumor-infiltrating DCs was corrected by intratumoral injection of FLT3-ligand-derived dendritic cells. The explanation for this difference was proposed to be the lack of production of the chemokines CCL4 and CXCL1 by β-catenin-deficient tumor cells.

Sema Kurtulus (Brigham and Women’s Hospital) presented data showing that TIGIT expression is increased on CD8 T cells and Tregs in tumor tissue. TGIT is a co-inhibitory molecule whose ligation leads to increased IL10 production and decreased IL12 production by DCs, and inhibition of T-cell proliferation and cytokine production. In their murine model, TIGIT marked a subset of Tim-3^+^ PD-1^+^ CD8^+^ T cells that were most dysfunctional. B16 melanoma and CD38 colon carcinoma cells grew slower in TIGIT-deficient mice. CD8 T cells from these mice proliferated better and produced more granzyme B but their transfer to wild type tumor-bearing mice did not decrease tumor growth. However, if Tregs were TGIT deficient, the antitumor response was enhanced upon transfer of CD8 T cells. Thus, TIGIT appears to suppress antitumor immune responses by inhibiting CD8 T cells. The blockade of TIGIT appears to slow tumor growth modestly but maximal decreases in tumor growth were demonstrated when it was combined with TIM-3 blockade. Together, TIGIT appears to be another immune checkpoint to consider in combined immunotherapy approaches for cancer.

Marcela Maus (University of Pennsylvania) reported on the development of a humanized chimeric antigen receptor (CAR) that recognizes the variant III mutation of the epidermal growth factor receptor (EGFRvIII). EGFRvIII, which has an in-frame deletion of a portion of the extracellular domain that produces a neoantigen, is found in a number of different malignancies, particularly glioblastoma multiforme. They selected a single chain Fv specific for the mutant EGFRvIII peptide and incorporated it into a second generation CAR T cell that includes the 4-1BB costimulatory and T-cell receptor zeta chain signaling domains. T cells transduced with the CART-EGFRvIII killed antigen-bearing targets, and proliferated and produced cytokines specifically to EGFRvIII. There was no cross-reactivity with wild type EGFR in vitro. In vivo, CART - EGFRvIII failed to recognize normal human skin implanted in immunodeficient mice whereas cetuximab-based CAR T cells reacted both to normal skin and EGFRvIII. The authors demonstration that CAR T cells could control tumor growth in xenogeneic subcutaneous and orthotopic models of human EGFRvIII positive glioblastoma led to the initiation of a phase I trial in which patients with EGFRvIII^+^ glioblastomas will receive CART-EGFRvIII alone or in combination with temozolomide after surgery.

### Immunoscore update

Bernard A. Fox (Earle A. Chiles Research Institute) opened the session with the bold statement that “the immune system is the ‘agent’ that improves outcome and CURES people with metastatic cancer”. Citing the historical efficacy of IL-2 and the appearance of long-term responders after treatment, first with anti-CTLA-4 and now with anti-PD-1, there is growing evidence to support this concept. Nevertheless, the majority of patients with solid tumors do not experience long-term remissions following immunotherapy. Based on work done by Jerome Galon and colleagues in patients with colon cancer, which suggested that type, density and location of immune cells within the tumor may be more predictive of clinical outcome than standard TNM staging, the SITC Immunoscore Task Force was established [4]. The goals of the Task Force are to validate the immunoscore as a prognostic biomarker, and if appropriate, to modify the relevant AJCC TNM classification. These approaches will likely lead to significant progress in the histopathologial assessement of malignant disease, including the quantification of immune cell infiltrates. Dr. Fox mentioned the members of the steering committee and described the preliminary work to engage centers throughout the world in this important initiative. As of November 2014, control slides have been stained and imaged. Ongoing work includes uploading images and adopting methods for analyzing images and determining appropriate cut-off values and performing statistical analysis. The choice of markers for the immunoscore panel will evolve as new data arise. The constantly improving technology allows for analysis of multiple markers, thereby complicating the analysis but broadening the potential value of this approach. The work needs to have substantial bioinformatics infrastructure. The SITC website has the slides showing the impressive analysis that is now possible using this strategy.

### Coinhibition and costimulation: targets and strategies

Brendan Curti (Earle A. Chiles Research Institute) started the session entitled “Coinhibition and Costimulation: target and strategies”. He shared clinical data concerning a phase I trial using a mouse anti-OX40 antibody as an immune-stimulatory agent in late stage cancer patients. OX40 is a TNF receptor family member expressed on CD8 T cells, and more strongly on both conventional CD4 T cells and T regulatory CD4 T cells. Expression of its ligand (OX40L) is only transient on antigen presenting cells (APCs). OX40 agonistic antibody was shown to costimulate CD4 T cells to increase memory development of Th1 and Th2 T cells. The treatment had low to mild toxicity and with only one treatment cycle, a third of the patients showed some degree of tumor regression in at least one metastatic lesion. Binding of the antibody was confirmed on CD8 and on CD4 T cells, following the pattern of expression of OX40 on these cells. This binding correlated with increased proliferation of CD4 T cells, CD8 T cells and NK cells. Stabilization of the disease in some patients was associated with proliferation of conventional CD4 and CD8 T cells expressing the activation markers HLA-DR and CD38. Furthermore, when these cells were tested against autologous tumor, cells from 2 out of 3 patients responded by producing increased levels of IFN-γ. Another phase I protocol using a Fc-OX40L fusion molecule was opened in September 2014 following these encouraging results. Dr. Curti also proposed to use OX40 stimulation in combination with checkpoint inhibitors, costimulatory antibodies, or radiation therapy.

Kavita Dhodapkar (Yale University) presented a study comparing transcriptomic data of T cells and monocytic cells obtained from patients before and after treatment with anti-CTLA4 or anti-PD-1 alone or in combination*.* She showed that the transcriptomic changes observed after anti-PD-1 treatment were associated with a NK cell signature, with an increased level of granzyme B expression. Interestingly, the changes observed after anti-CTLA4 treatment were different, with a strong association with proliferation, and in particular an increase in Ki67 expression. The combination of both antibodies had a synergistic effect on T cells with increased granzyme B and Ki67 expression, as well as production of IFN-γ and IL-2 within TILs and PBMCs.

Ines Pires da Silva (NYU Cancer Center) presented data associating NK cell exhaustion with melanoma progression. NK cells from PBMCs of melanoma patients showed decreased capacity to produce IFN-γ and to proliferate. This correlated with an increased expression of Tim-3 on NK cells. Blocking of Tim-3 with an antibody restored IFN-γ production and proliferation of NK cells coming from patients.

As last speaker of the session, Robert Vonderheide (University of Pennsylvania) presented pre-clinical and clinical data concerning the use of the agonistic anti-CD40 antibody in cancer immunotherapy. CD40 is a critical regulator of the adaptive and innate immune systems, playing a central role in the licensing of dendritic cells for stimulating antigen-specific T cells. In a mouse model of pancreatic adenocarcinoma, he showed previously published data demonstrating that combination of anti-CD40 antibody together with chemotherapy could induce tumor regression mediated by macrophages. Addition of checkpoint inhibitors further increased tumor regression. In patients suffering from advanced pancreatic ductal adenocarcinoma, a phase I study using similar treatment showed mild toxicity and some tumor responses. In one melanoma patient that received anti-CD40 treatment and experienced 9 year-complete remission, a drastic change in the TCR repertoire of T cells was found, highlighting that anti-CD40 treatment can also affect T cell development. In conclusion, anti-CD40 treatment is a suitable candidate for neoadjuvant therapy for pancreatic cancers, with manageable toxicity and good results.

### Adoptive immunotherapy

In the “adoptive immunotherapy” session, Patrick Hwu (University of Texas, MD Anderson Cancer Center) showed that failure to respond to checkpoint inhibition therapy was not indicative of a bad response to adoptive cell therapy (ACT), as 29% of the non-responders to checkpoint blockade therapy had a measurable response to ACT. A detailed comparison was performed of samples from patients that have successfully undergone ACT with TIL (representing 44% of the patients) with the ones for whom this therapy was not successful. A nanostring study using paraffin samples showed a correlation between the high expression of T cell activation markers (including “checkpoint” inhibitory receptors) to a favorable outcome after ACT. To further increase ACT efficiency, Dr. Hwu proposed to combine TIL therapy with the use of BRAF inhibitors, as it was shown that BRAF inhibition enhances T cell infiltration of metastatic melanoma lesions. Another possible combination is the use of PI3K inhibitors. In many tumor types, PTEN loss induces increased PI3K signaling. Dr. Hwu showed that these tumors are more resistant to T cell infiltration, associated with decreased patient survival. Furthermore, he found that when the PI3K pathway is more active in a patient’s tumor, there is less chance that TILs are prone to be expanded *in vitro*. High PI3K signaling in vivo was also associated with poor cytolytic function. One possible mechanism could be the intra-tumoral downregulation of the autophagy pathway. Finally, he also suggested a possible combination of ACT/PI3K inhibitor with PD-1 blockade.

Per thor Straten (Centre for Cancer Immune Therapy Copenhagen) summarized the results obtained after ACT in melanoma patients in his center. They obtained a 20% complete response rate in 31 patients despite a lower dose of IL-2 during the treatment, with a remarkable 92% success rate for establishing TIL cultures. Taking advantage of these TILs, they attempted to decipher the epitope specificity of T cells using 145 tetramers synthesized with known MHC class I cancer epitopes, mostly non-mutated ones. The total of all these epitopes only represented a low percentage among the cultured TILs, indicating that most of the recognized epitopes in these cultures remain undefined.

Eric Tran (NIH/NCI) presented the results obtained for ACT in advanced cholangiocarcinoma, showing poor efficiency for inducing clinical responses. However, the study of one case suggested that highly enriched TILs recognizing a mutated epitope were effective at inducing tumor regression. In an attempt to extend this investigation to other patients, Dr. Tran and colleagues systematically sequenced the genome of the tumors from each patient to identify potential mutated epitopes recognized by T cells. Several mutations were then incorporated to minigenes used to transduce APCs. These APCs were used to stimulate the patient’s TILs. On amplified cultures, multiple epitopes encoded by the minigenes were separated on several peptides used again to amplify TILs. Reactive TIL populations were successfully expanded from 7 out of 8 patients. These cultures will be used to treat patients.

Another study was presented which focused on metabolic switches. With the objective to enhance the capacity of amplified TILs to treat patients, Madhusudhanan Sukumar (NCI) studied the metabolic profile of tumor-specific CD8^+^ T cells. He summarized the results from two studies during the meeting. One concerned the switch from oxidative phosphorylation to aerobic glycolysis, once a T cell switches from a quiescent state to an activated one. He showed that decreasing the glycolysis by using a pharmacological inhibitor (2-deoxyglucose) induced “better” differentiation of memory T cells and improved their immunotherapeutic potential against cancer. The second part focused on the mitochondrial status of the T cells. Dr. Sukumar demonstrated that a low membrane potential was associated with a much better capacity to expand *in vivo*, the cells being a hematopoietic precursor like Lin- Sca-1+ cKit + (LSK) cell or a T cell. This was associated with the expression of the transcription factors Bcl6, Tcf7 and Klf2. The cells with a high membrane potential showed poor capacity to expand *in vivo*, and associated with high level of Eomes. One possible explanation for this defect in expansion/survival, may be a higher level of reactive oxygen species in the cells related to a decrease in the transcriptional program encoding for the anti-oxidative machinery.

The session ended with two presentations about CAR T cells. The first one was given by Michael Jensen (University of Washington), who presented several strategies that his group developed to enhance CAR T cell efficiency, optimizing the type of cells used, the purification protocols and the nature of the vector. He proposed approaches that take advantage of the integration of the mutated form of the EGFR (EGFRt) in the construct to purify the CAR-expressing T cells and also by potentially targeting this molecule in the patient by using cetuximab to eliminate the CAR T cells in case of off-target toxicity.

Michel Sadelain (Memorial Sloan Kettering Cancer Center) presented the results of a phase I study using the CD19-specific 19-28z CAR to treat B cell acute lymphoblastic leukemia. Among 16 patients, the overall complete response rate was very high (88%), with a time to complete response averaging 24.5 days and allowing the transition of most of these patients to a standard-of-care allogeneic hematopoietic stem cell transplant. In these patients, he showed that the initial tumor burden strongly correlated with the possibility to develop severe cytokine-release syndrome, and showed several strategies to manage this adverse effect.

In summary, the meeting was very rich in novel results and concepts for improving immunotherapy of cancer. It provided ample opportunities for SITC members and visitors to connect, communicate and enforce existing collaborations and embark on new ones. The meeting revealed the leading role SITC plays as an innovator in the field, where representatives of academia and industry meet for promoting progress in cancer research and development of novel therapies.
